# Development of a simple multiple mutation detection system using seed-coat flavonoid pigments in irradiated Arabidopsis M_1_ plants

**DOI:** 10.1038/s41598-022-26989-z

**Published:** 2022-12-28

**Authors:** Satoshi Kitamura, Shoya Hirata, Katsuya Satoh, Rie Inamura, Issay Narumi, Yutaka Oono

**Affiliations:** 1 Project “Ion Beam Mutagenesis Research”, Department of Radiation-Applied Biology Research, Takasaki Advanced Radiation Research Institute, National Institutes for Quantum Science and Technology, Takasaki, Gunma 370-1292 Japan; 2 Graduate School of Life Sciences, Toyo University, Itakura, Gunma 374-0193 Japan

**Keywords:** Plant breeding, Plant genetics

## Abstract

Ionizing radiation induces genetic variations in plants, which makes it useful for plant breeding. A theory that the induced mutations occur randomly in the genome has long been accepted, but is now controversial. Nevertheless, a comparative analysis of the mutations at multiple loci has not been conducted using irradiated M_1_ genomes that contain all types of mutations. In this study, we identified Arabidopsis mutants (*pab2* and *pab3*) in a mutagenized population of an anthocyanin-positive seed mutant (*ban*). Both *pab2* and *pab3* were revealed to be double mutants (*tt4 ban* and *tt8 ban*, respectively) that produced similar anthocyanin-less immature seeds, but differentially colored mature seeds. These features enabled the seed color-based detection of de novo M_1_ mutations in *TT4* or *TT8* following the irradiation of double heterozygous plants (*TT4*/*tt4 TT8*/*tt8 ban*/*ban*). Most of the irradiated double heterozygous plants produced anthocyanin-positive immature seeds, but 19 plants produced anthocyanin-less immature seeds. Of these 19 mutants, 2 and 17 exhibited *tt4*- and *tt8*-type mature seed coloration, respectively. The molecular analysis of the seed coat DNA from randomly selected anthocyanin-less seeds detected mutations at the locus predicted on the basis of the phenotype. Thus, the simple system developed in this study can reliably detect radiation-induced mutations at multiple loci in irradiated Arabidopsis M_1_ plants.

## Introduction

Genetic variations, which are major factors contributing to the diversity and evolution of all organisms, have long been used to enhance cultivated plant lineages. Artificial mutagenesis induces genetic variations and is frequently used in plant breeding programs. Ionizing radiation is one of the most common mutagens in these programs because the associated methods are relatively simple and are applicable for all plant materials and species^1–3^. Because radiation tracks are allocated randomly in the target nuclei, the radiation-induced DNA damages likely occur randomly in the genome^4,5^. However, repair of the damaged DNA could not be equally proceeded at respective regions in the genome. For example, it is well known that structurally altered DNA lesions are excised and repaired more effectively in transcribed regions than un-transcribed regions^6^. It was also reported that DNA double-strand breaks in transcriptionally active regions was accurately repaired by error-free homologous recombination pathway in human cells^7,8^. Configuration of chromatin at DNA lesions is also important for determining their repair efficiency^9^. Very recently, using large datasets of genomics and epigenomics in Arabidopsis, spontaneous mutations were shown to occur non-randomly in the genome^10^. Despite of the recent progress, a long-standing theory on the randomness of radiation-induced mutations in the genome has not been fully verified in higher plants. If the radiation-induced mutations occur in non-random, more efficient mutation breeding program might be explored.

Mutation analysis in irradiated M_1_ plants is important for addressing directly mutagenic effects of radiations, and the obtained knowledge would be helpful to plant breeding especially for vegetatively propagated plants. Radiation generates unique mutational patterns in the cells of irradiated tissues. Thus, the irradiated M_1_ plants comprise a mixture of cells with varying mutational patterns. Because of the complexity of M_1_ plants, radiation-induced mutations have generally been analyzed in the self-pollinated plants derived from the irradiated M_1_ plants (i.e., M_2_ or subsequent generations)^11,12^. However, some types of radiation-induced mutations, including large deletions and chromosomal rearrangements, are hardly transmitted from one generation to the next^13^. Thus, to accurately evaluate the randomness of the mutations induced by ionizing radiation, the irradiated M_1_ plants that harbor all types of mutations should be used for developing a simple experimental system that enables a comparative analysis of mutations at multiple genomic loci.

Several heterogenous marker systems have been used to detect mutations in mutagenized M_1_ plants. The β-glucuronidase-encoding gene (*GUS*) is a typical example. Constructs for the functional silencing of *GUS* are introduced into the target genome prior to mutagenesis. When a mutation restores *GUS* expression, cells harboring the restored *GUS* in mutagenized plant tissues are detected on the basis of blue pigmentation^14^. Several *GUS* constructs designed to detect specific types of DNA changes, such as base substitution and homologous recombination, have been used^15,16^. A single genome may simultaneously contain several *GUS* constructs, but all of the constructs will produce the same blue pigmentation when at least one of them restores *GUS* expression. Accordingly, it is difficult to discriminate between mutations at multiple loci in a single plant via phenotypic examinations. Another heterogenous marker gene, *rpsL* (encoding the ribosomal S12 protein in *Escherichia coli*), has also been used to detect mutations in the M_1_ genome. Because mutations in *rpsL* result in streptomycin-resistant *E. coli*, plasmids rescued from transgenic plants with *rpsL* in their genome are inserted into *E. coli* cells, which are screened on the basis of streptomycin resistance and analyzed to reveal mutations in the *rpsL* region^17^. In contrast to *GUS* constructs, a single *rpsL* construct can detect multiple types of mutations, including single base substitutions and insertions/deletions (indels), if they occurred within its genic region (375 bp). However, when multiple *rpsL* constructs are introduced into one genome, it is difficult to determine the original genomic location of the mutated *rpsL* gene. Therefore, although these systems can provide researchers with valuable information regarding the mutations in plant genomes caused by ionizing radiation^18,19^, they are not suitable for detecting a wide range of radiation-induced mutations at multiple loci in a single plant.

Flavonoid pigments are conserved in many plant species and are frequently used to detect spontaneous and artificially-induced mutations^20,21^. The flavonoid biosynthetic pathway in the model plant Arabidopsis has been extensively analyzed at the molecular level. All of the flavonoid compounds in Arabidopsis are synthesized in a single biosynthetic pathway, and the genes encoding the enzymes catalyzing the pathway reactions have been identified^22^. The brown coloration of mature Arabidopsis seeds is due to the oxidation of the non-colored proanthocyanidins^23^. The disruption of any of the biosynthetic steps in this pathway decreases the accumulation of brown pigments in the seed coat, resulting in the so-called *transparent testa* (*tt*) phenotype. Notably, mature seeds of some *tt* mutants are differentially colored, making them visually distinguishable^24^. This indicates that each *tt* mutant can be discriminated according to the color of the mature seeds. Another important feature is that the flavonoid biosynthetic pathway in Arabidopsis consists of only one set of genes in the genome. This enables the detection of mutations in the flavonoid biosynthesis-related genes in M_1_ plants on the basis of the loss of heterozygosity (LOH). The detection of LOH in the M_1_ generation would also decrease the number of plants that must be analyzed compared with the detection of homozygous mutants in the M_2_ or later generations. In this study, we tried to develop a system for detecting mutations at multiple loci in the M_1_ generation by examining flavonoid pigmentation in Arabidopsis seeds. The discrimination of two mutants according to seed color was supported by the detection of mutations in the genes responsible for the phenotype. Using this system, we revealed that the mutation frequencies differed significantly at two loci (*TT4* and *TT8*).

## Results

### Isolation and characterization of *pab2* and *pab3* mutants

**Figure 1:**
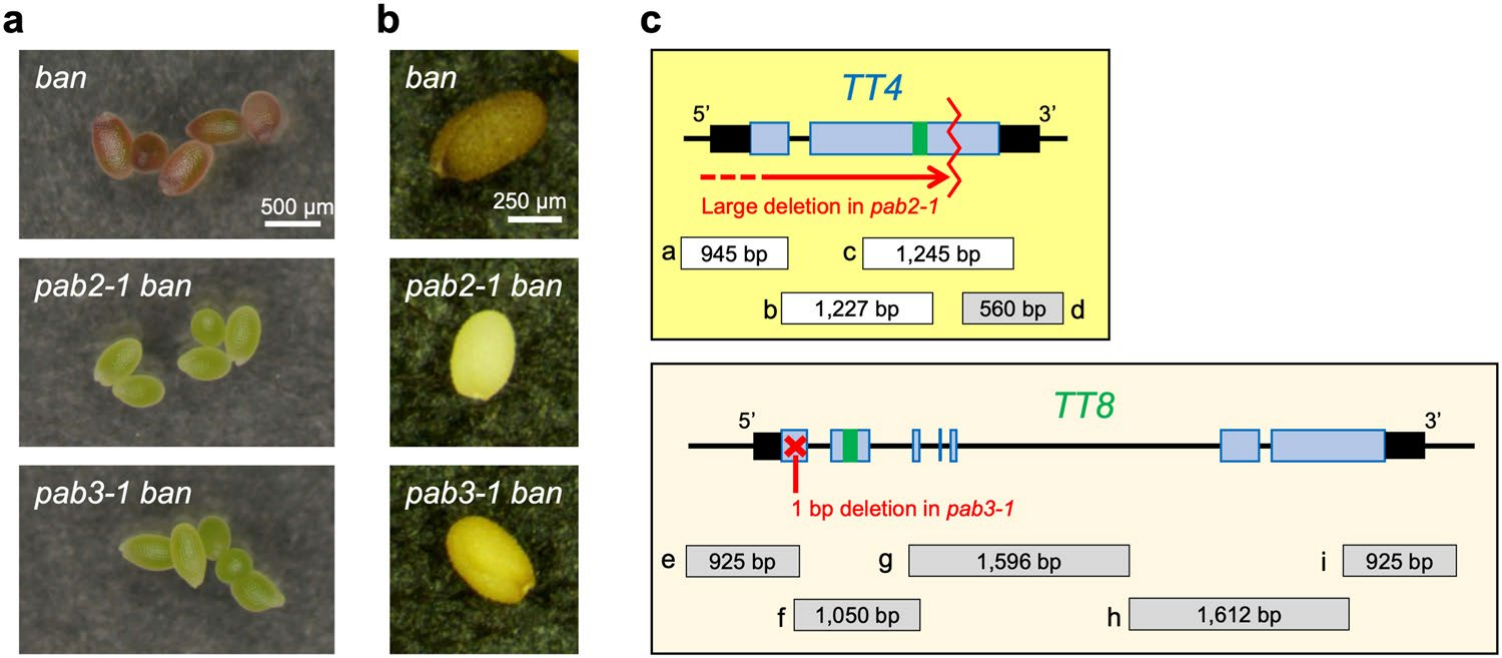
Characterization of *pab2-1 ban* and *pab3-1 ban*. Seed color at the immature (**a**) and mature (**b**) stages in *ban*, *pab2-1 ban*, and *pab3-1 ban*. (**c**) Schematic representation of mutations in the *TT4* and *TT8* regions for *pab2-1 ban* (top) and *pab3-1 ban* (bottom), respectively. Pale blue and black boxes represent exons and untranslated regions, respectively. Mutations in *pab2-1 ban* and *pab3-1 ban* are indicated in red. Green bars indicate the region used for the qPCR analysis. Gray and white boxes below the gene (labeled with a–i) indicate the amplified and non-amplified fragments, respectively, for the PCR performed using site-specific primers and template DNA from the *pab2-1 ban* and *pab3-1 ban* mutants.

Using a mutagenized M_2_ population derived from *banyuls* (*ban*) plants, we previously isolated a mutant with a *pale-ban* (*pab*) phenotype, which was designated as a *pab1* mutant^25^. The subsequent screening of this population detected two new *pab* mutants (*pab2-1* and *pab3-1*), both of which had immature seeds with relatively low anthocyanin contents (Fig. 1a). Because the *pab2* and *pab3* phenotypes were only detected in the *ban* background, we referred to these mutants as *pab2 ban* and *pab3 ban*, respectively. The results of the rough-mapping analysis for the *pab2-1 ban* and *pab3-1 ban* mutants indicated that the genes responsible for their strong anthocyanin-less phenotypes were located near the *TT4* and *TT8* loci, respectively. The *TT4* gene encodes the first enzyme of the flavonoid biosynthetic pathway^26^, whereas the *TT8* gene encodes a seed coat-specific transcription factor that is necessary for the production of the “late” enzymes of the pathway^27^. The examination of the sequences in the *TT4* and *TT8* regions revealed mutations in these two genes. More specifically, a large fragment, including most of the *TT4* region, was deleted in *pab2-1 ban* (Fig. 1c). In *pab3-1 ban*, a single nucleotide deletion (T146∆) was detected in *TT8*, which resulted in a truncated tt8 protein that contained only 58 amino acids, making it considerably shorter than the wild-type TT8 protein, which comprises 518 amino acids (Fig. 1c).

At the immature seed stage, the phenotypes of the *pab2-1 ban* and *pab3-1 ban* mutants were similar, but they obviously differed from the phenotype of the original *ban* mutant (Fig. 1a). However, there were clear differences in the *pab2-1 ban* and *pab3-1 ban* mature seeds, which were pale yellow and pale brown, respectively (Fig. 1b). The phenotypic features of the immature and mature seeds prompted us to develop a new mutation detection system for the M_1_ plants as described in the following section.

### Development of a mutation detection system for mutagenized plants using double heterozygotes for *TT4* and *TT8*

**Figure 2:**
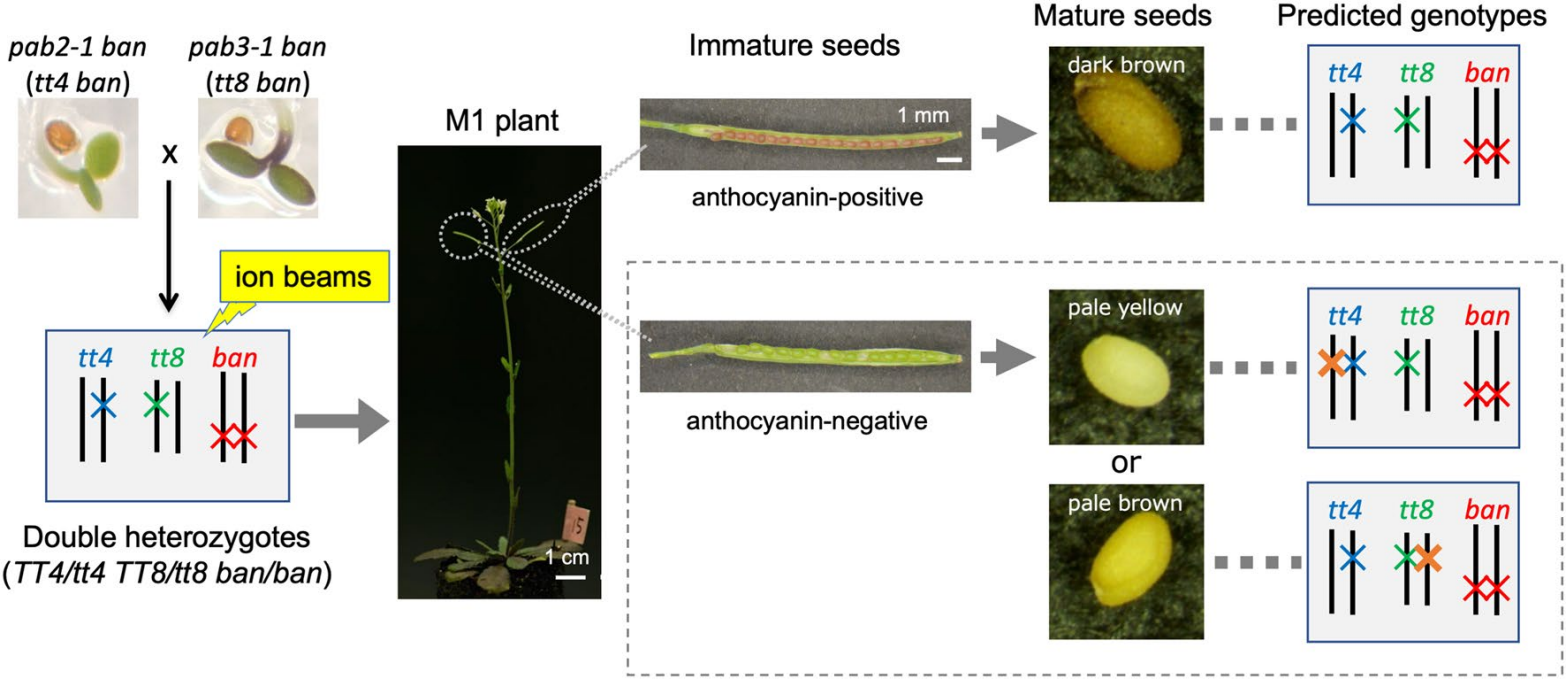
Strategy for detecting mutations at multiple loci in the M_1_ generation. The cross-pollination of *pab2-1 ban* and *pab3-1 ban* generated double heterozygous plants for *TT4* and *TT8*. The double heterozygotes were irradiated with ion beams. When the wild-type allele at the heterozygous *TT4* or *TT8* locus was mutated by irradiation, anthocyanin-less immature seeds were produced by the tissues with the mutated *tt4* or *tt8* allele in the M_1_ plants. These seeds were easily discriminated from the red immature seeds produced by the tissue with non-mutated *TT4* and *TT8* alleles. The M_1_ plants producing anthocyanin-less immature seeds were grown until the seeds matured to determine whether their mature seeds were pale yellow (*tt4*-type) or pale brown (*tt8*-type).

The cross-pollination of the *pab2-1 ban* and *pab3-1 ban* mutants generated plants with a double heterozygous genotype for *TT4* and *TT8* in the *ban* background (genotype: *TT4*/*tt4 TT8*/*tt8 ban*/*ban*). The double heterozygous plants had a phenotype that was similar to that of the *ban* single mutant (e.g., red immature seeds and dark brown mature seeds). A deleterious mutation in the wild-type allele of the heterozygous *TT4* or *TT8* locus will result in a non-functional anthocyanin biosynthetic pathway. A mutated cell is expected to divide and form a mutant sector in the double heterozygous plant. When the sector harboring the deleterious mutation in the *TT4* or *TT8* locus contributes to seed coat formation (i.e., the terminal tissue of the mutated somatic cells), the seeds will have an anthocyanin-less phenotype. The anthocyanin-less seeds were clearly distinguished from the non-mutated anthocyanin-containing seeds at the immature seed stage (Fig. 2). Additionally, after the remaining mutant siliques matured and dried, the anthocyanin-less seeds were pale yellow (*tt4*) or pale brown (*tt8*), making it possible to easily differentiate between these seeds (Fig. 2). Thus, mutations at two distinct loci (*TT4* or *TT8*) were detectable on the basis of the seed color at the immature and mature stages.

The double heterozygous seedlings were mutagenized by irradiating them with carbon ions. The survival curve of the wild-type seedlings indicated that the shoulder dose (i.e., the dose at which the survival rate decreased sharply from nearly 100%) was about 30 Gy at 1 day after germination (DAG) (Supplementary Fig. S1). We selected 15 Gy (i.e., half the shoulder dose) for the mutagenesis of the double heterozygous seedlings with carbon ion beams. The irradiated double heterozygous plants were grown until flowering and seed formation. Immature seeds were removed from the young siliques of the irradiated plants and then their pigmentation levels were investigated using a stereomicroscope. None of the approximately 400 non-irradiated double heterozygous plants produced anthocyanin-less immature seeds. Of the 2588 double heterozygous seedlings irradiated with carbon ion beams, 19 produced anthocyanin-less immature seeds. The 19 candidate mutants showed strong phenotypes with nearly complete lack of anthocyanins, and candidates showing subtle reduced anthocyanins were not isolated. The candidate mutants showed normal morphology in general, except for the anthocyanin-less feature. We then compared the candidate mutants with the parental *pab2-1 ban* (*tt4 ban*) and *pab3-1 ban* (*tt8 ban*) plants in terms of the mature seed colors. Among the 19 candidate mutants, two had pale yellow *tt4*-type mature seeds, whereas the other 17 had pale brown *tt8*-type mature seeds (Table 1, Supplementary Fig. S2). If *TT4* and *TT8* were considered to be equally mutated (i.e., a probability for each *TT4* and *TT8* mutation is 0.5), this unequal distribution of *tt4* and *tt8* was significant (*p* < 0.001; two-sided binomial test). On the other hand, including the 5′- and 3′-untranslated regions, *TT8* comprises 4643 bp, which is about 2.6-times longer than *TT4*, which consists of 1789 bp. Taking the size difference into consideration (i.e., a probability for *TT4* mutation is assumed as 0.28 [1789 (1789 + 4643)^−1^]), distribution of two *tt4* and 17 *tt8* mutants was not significant (*p* > 0.06; one-sided binomial test).

**Table 1:** Screening of anthocyanin-less seeds produced by the double heterozygous plants irradiated with carbon ion beams.

	No. of plants observed	No. of plants having anthocyanin-less seeds	Pale yellow (*tt4*-type)	Pale brown (*tt8*-type)
Non-irradiation	430	0	0	0
irradiation	2588	19	2	17

### PCR analyses of the anthocyanin-less seed coat-enriched DNA

To confirm the presence of the de novo mutation responsible for LOH at the *TT4* and *TT8* loci in the anthocyanin-less seeds, we first tried to isolate seed coat-specific DNA by separating all of the endosperm and embryo cells from the seed coat, because the seed color is derived from the seed coat tissues, but not the endosperm and embryo^24^. However, it was difficult to obtain sufficient amounts of seed coat-specific DNA to conduct a subsequent analysis. This was likely because of the loss of seed coat cells during the dissection of a limited number of mutant seeds. Therefore, we decided to use seed coat-enriched samples, instead of seed coat-specific samples. To verify the quality of the obtained seed coat-enriched DNA, immature hybrid seeds produced by adding pollen with the *ban-4*/*ban-4* genotype (*ban-4* means the fourth allele of *ban*^28^) to the stigma with the *BAN*/*BAN* genotype were used as the model materials. In the model hybrids, the seed coat cells had the maternal *BAN*/*BAN* genotype, whereas the embryo cells had a mixture of maternal and paternal genotypes (*BAN*/*ban-4*). The PCR amplification of the *BAN* and *ban-4* fragments followed by the cleaved amplified polymorphic sequence (CAPS) analysis^29^ involving the restriction enzyme *Scr*FI, which digests the *BAN* allele, but not the *ban-4* allele, generated maternal *BAN* bands, but not the paternal *ban-4* band, for the seed coat DNA (Supplementary Fig. S3). This banding pattern was consistent with the predicted genotype and suggests that the obtained seed coat-enriched DNA was suitable for investigating the mutations responsible for LOH in anthocyanin-less immature seeds.

**Figure 3:**
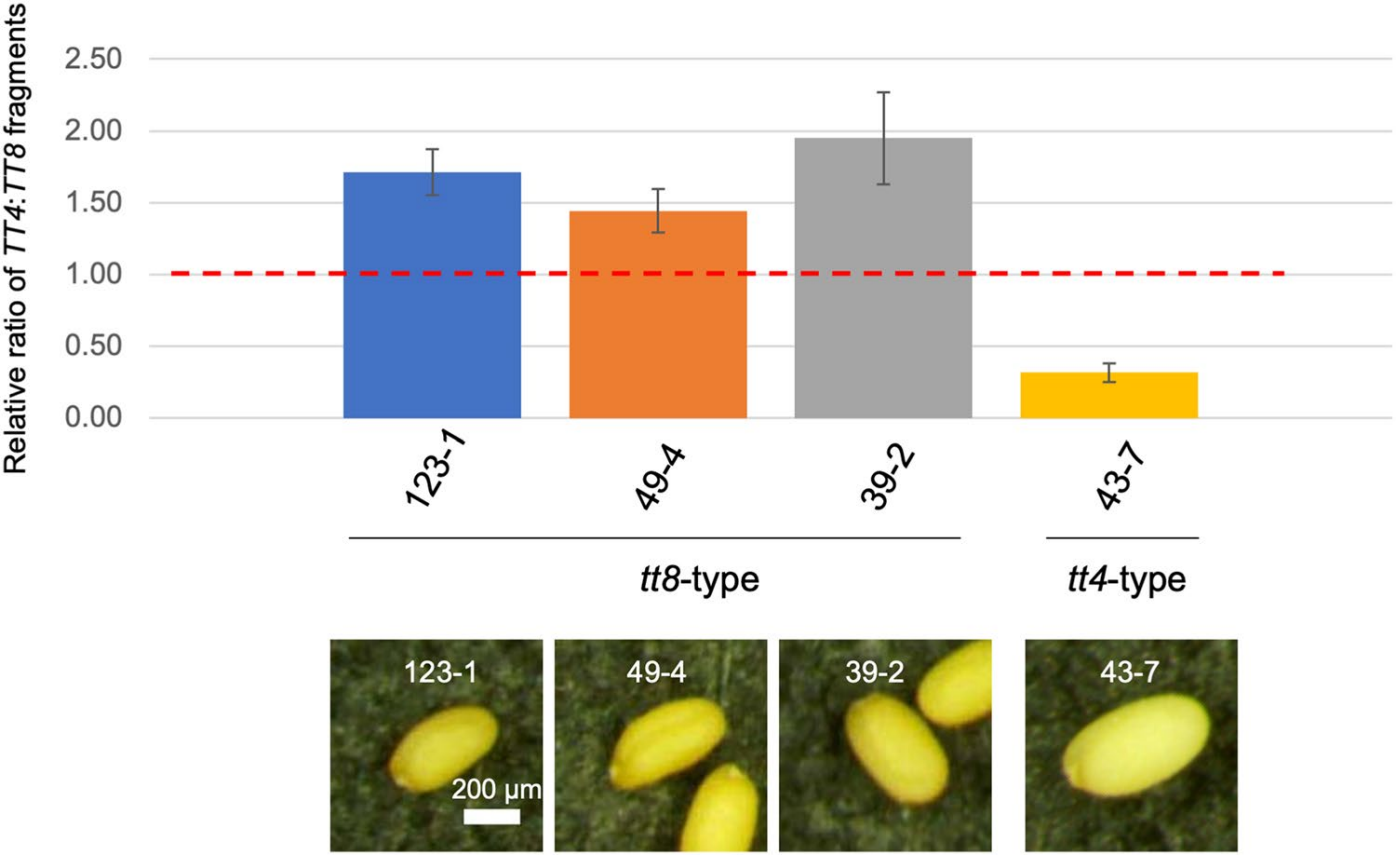
Quantification of the *TT4* and *TT8* fragments in DNA from anthocyanin-less seed coat-enriched tissues on the basis of a qPCR analysis. The *TT4*:*TT8* fragment ratios normalized against the corresponding ratio in the non-irradiated double heterozygous plants (red dotted line) are provided for the four mutants. Data are presented as the mean value and standard error from three biological replicates.

Four mutants with anthocyanin-less phenotypes were randomly selected at the immature seed stage for the dissection and examination of their seed coats. However, we were unable to determine whether these seeds were *tt4*- or *tt8*-type seeds. After the remaining seeds for the four selected mutants were allowed to mature and dry, three (123-1, 49-4, and 39-2) and one (43-7) mutants were revealed to produce *tt8*- and *tt4*-type seeds, respectively. Therefore, we expected that the mutations responsible for LOH would be found at the corresponding loci in the four mutants. We first examined the possibility of small mutations at the *TT4* and *TT8* loci by performing a site-specific PCR amplification and then analyzing the amplicon sequences. All four mutants produced amplification patterns for the *TT4* and *TT8* regions (e.g., Supplementary Fig. S4) that were similar to those of the control DNA (i.e., from non-irradiated double heterozygous plants). The sequencing of the amplified fragments did not detect any mutations responsible for LOH in *TT4* and *TT8*. This was probably due to the contamination of the seed coat-enriched tissues with other non-mutated cells (e.g., endosperm cells), and this explanation was supported by the results on quantitative real-time PCR and dosage analyses (see below). We next addressed another possibility that deletions larger than the amplified fragment occurred at the *TT4* or *TT8* locus. Such a deletion would be difficult to detect during a site-specific PCR analysis. Accordingly, a quantitative real-time PCR (qPCR) analysis of the *TT4* and *TT8* regions was performed, with the ratio of the quantity of the *TT4* fragments to that of the *TT8* fragments normalized against the corresponding ratio for the non-irradiated double heterozygous plants. The relative *TT4*:*TT8* fragment ratio would be less than 1 if a mutant lacked *TT4*, but contained *TT8*. Conversely, the relative *TT4*:*TT8* fragment ratio would be greater than 1 if a mutant lacked *TT8*, but contained *TT4*. The qPCR analysis indicated that three mutants (123-1, 49-4, and 39-2, all of which exhibited the *tt8*-type phenotype) had a relative *TT4*:*TT8* fragment ratio of approximately 1.5 or higher, whereas the other mutant (43-7, which exhibited the *tt4*-type phenotype) had a relative ratio of approximately 0.25 (Fig. 3). These results suggest that the three pale brown *tt8*-type mutants and the one pale yellow *tt4*-type mutant harbor deletion-type mutations in *TT8* and *TT4*, respectively. These findings were in accordance with our phenotype-based discrimination.

The target sequence used for the qPCR analysis of *TT4* was present in the wild-type *TT4* allele, but not in the *pab2-1* allele (Fig. 1c). Nevertheless, some *TT4* fragments were detected in the *tt4*-type mutant seed coat (43-7), reflecting the presence of wild-type *TT4* fragments in the seed coat-enriched DNA. This was consistent with the detection of non-mutated wild-type *TT4* DNA fragments during the site-specific PCR analysis of the mutant DNA (Supplementary Fig. S4) that might include small amount of other non-mutated cells like endosperm cells.

### Dosage analysis of the anthocyanin-less seed coat-enriched DNA

**Figure 4:**
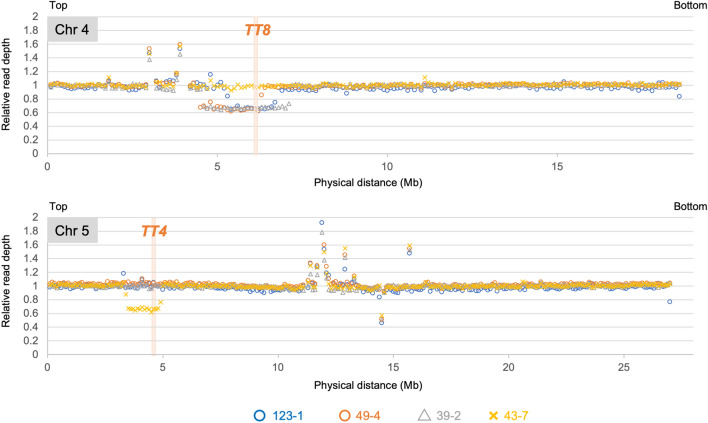
Dosage variations on chromosomes 4 and 5 for the four anthocyanin-less seed coat-enriched DNA samples (123-1 (blue circles), 49-4 (orange circles), 39-2 (gray triangles), and 43-7 (gold crosses)). The approximate positions of the heterozygous *TT8* and *TT4* loci are indicated by pale orange bars on chromosomes 4 and 5, respectively.

To generate additional evidence of the large deletions at the heterozygous *TT* loci, genome-wide DNA fragment dosage variations were evaluated using the seed coat-enriched DNA. Short reads obtained by next-generation sequencing were mapped to the Arabidopsis reference genome, and the reads in non-overlapping 100-kb chromosomal bins were counted. The relative read depth (RRD) was calculated by dividing the average read depth for the respective bins by the median depth for five chromosomes in each line. When RRD was plotted to the corresponding chromosomal region, the large deletions were depicted as consecutive plots with RRD values that were lower than those in non-deleted regions that had a theoretical RRD of 1. For the *TT8* locus on chromosome 4, one mutant (43-7) had an RRD value of approximately 1, whereas the other three mutants (123-1, 49-4, and 39-2) had an RRD value of approximately 0.65 (Fig. 4, top). In contrast, for the *TT4* locus on chromosome 5, one mutant (43-7) had an RRD value of approximately 0.65, whereas the other three mutants (123-1, 49-4, and 39-2) had an RRD value of 1 (Fig. 4, bottom). Because each plot in Fig. 4 represents 100 kb, the regions with low RRD values appeared to be extremely large (i.e., Mb-order) in the four anthocyanin-less seed coats (1.5, 1.9, 2.6, and 1.5 Mb in 123-1, 49-4, 39-2, and 43-7, respectively). Hence, the three *tt8*-type mutants (123-1, 49-4, and 39-2) and the one *tt4*-type mutant (43-7) have de novo mutations responsible for the LOH at either the *TT8* or *TT4* locus. Moreover, all of these mutations were large deletions. This was consistent with the qPCR data as well as the phenotype-based discrimination (Fig. 3).

## Discussion

To date, comparing mutation frequencies at multiple loci in the M_1_ genome has been challenging. We recently used the LOH of three anthocyanin biosynthesis-related genes to detect genome-wide mutations in gamma-irradiated M_1_ Arabidopsis leaves^30^. In this earlier study, an anthocyanin-less sector formed in the leaves of the irradiated M_1_ plants when one of the wild-type alleles at three heterozygous loci was mutated. However, unlike the current study, the mutated locus causing the anthocyanin-less phenotype was not identified on the basis of the observed phenotype alone because visible characteristics, including color intensity, were not distinguishable among the LOH of the three genes. In the present study, we focused on the differences in the colors of mature Arabidopsis seeds, which revealed that mutations in *TT4* and *TT8* can be clearly distinguished by simply examining the seed phenotype of irradiated double heterozygous M_1_ plants. The introduction of the *ban* mutation in the genetic background facilitated the screening for LOH at the immature seed stage (Fig. 2). This enabled the screening of seed color mutants from multiple inflorescence stems in a single M_1_ plant. The mutation frequency, which was calculated as the number of plants that produced anthocyanin-less seeds/total number of examined plants, was 0.7% (two *tt* genes as targets in this study). This was substantially higher than the 0.06% mutation frequency in the previous screening of anthocyanin-less stems of Arabidopsis M_2_ plants (seven *tt* genes as targets)^11^. Thus, the method used in the current study can effectively detect mutations. Additionally, screening at the immature seed stage allowed for the extraction of genomic DNA from fresh seed coats. The analyses of the seed coat-enriched DNA indicated that the four selected mutants have deletion-type mutations in either *TT4* or *TT8* (Figs. 3 and 4). The results were consistent with the phenotype-based predicted mutations, confirming the reliability of the mutation detection system developed in this study.

In the mutation detection system developed here, candidate mutants were screened based on the visible phenotypes in immature seeds (i.e., differences in pigmentation of the immature seeds) (Fig. 2). It should be noted that all candidate mutants isolated in this study showed nearly complete loss of anthocyanins in immature seeds, and candidates showing subtle reduced pigmentation were not isolated. It is theoretically possible to isolate mutants with weak phenotypes, because the immature seed does not contain any other pigments, and in fact, we previously isolated a mutant with pale pink to orange pigmentation from a mutagenized M_2_ population of *ban* plants^25^. Subsequent molecular analysis of the four randomly selected mutants unraveled that they harbored extremely large deletions (> 1.5 Mb) including heterozygous *TT* loci (Fig. 4). It is obvious that a large deletion has more chance to change a function of *TT4* or *TT8* than other types of mutations such as single base substitution, small indel, and chromosomal rearrangement whose breakpoints were located within an intragenic region^13^. Thus, it would be reasonable that large deletions are the majority of mutations responsible for LOH, although mutations other than large deletions might occur at the *TT* loci in the remaining candidate mutants. On the other hand, a combination of site-specific PCR, qPCR and dosage analysis used in this study would be difficult to identify the breakpoints of inversions and translocations within the intragenic region. To detect any types of mutations at the heterozygous *TT* loci including junctions of structural variations, whole-genome resequencing using seed coat-enriched DNA would be effective, as done in the previous report^30^.

The qPCR analysis revealed that all four analyzed mutants have large deletions in *TT4* or *TT8* (Fig. 3). The qPCR data were verified by the dosage analysis, which demonstrated that large deletions were responsible for LOH in all four examined mutants, and that all of the deletions at *TT* loci were extremely large (≥ 1.5 Mb) (Fig. 4). Consistent with this finding, we recently detected the LOH of three anthocyanin biosynthesis-related genes in gamma-irradiated Arabidopsis M_1_ leaves, and observed that the lack of anthocyanin in four of five leaves was due to extremely large deletions (0.5–5.0 Mb), whereas the anthocyanin-less phenotype of one leaf was caused by a small mutation at one of the three heterozygous loci^30^. In contrast to the mutations in the M_1_ genomes causing the anthocyanin-less phenotypes, large mutations (i.e., deletions) and small mutations (i.e., single base substitutions and small indels) are reportedly responsible for anthocyanin-less phenotypes at similar frequencies in Arabidopsis M_3_ mutants induced by carbon ion beams^31^. Focusing on the large deletions, 30 large deletions (≥ 149 bp) were detected by array comparative genomic hybridization in 13 Arabidopsis M_3_ mutants induced by argon and iron ion beams, but an extremely large deletion (> 1 Mb) was not found^32^. Similarly, out of 22 large (≥ 100 bp) deletions found in 16 Arabidopsis M_3_ mutants induced by argon and carbon ion beams, single extremely large deletion (> 1 Mb) was found^33^. Because extremely large deletions in the M_1_ genome will hardly be transmitted to the next generation^13^, the number of extremely large deletions affecting the phenotype will decrease in the M_2_ or later generations. Thus, substantial number of large deletions, which are induced more frequently by ionizing radiation than by chemical mutagens such as ethyl methanesulfonate^2^, have not been effectively detected during standard mutation analyses of genomes from the M_2_ or later generations, but they can be explored more thoroughly by analyzing the M_1_ genome. Considering it is useful for detecting a wide range of mutations^30^, an analysis of the M_1_ genome is also important for accurately determining the occurrence of mutations in plant genomes induced by ionizing radiation.

On the basis of the mutation detection system developed in this study, the *tt4* phenotype (two plants) was 8.5-times less frequently observed than the *tt8* phenotype (17 plants) following the carbon ion irradiation (Table 1). The unequal distribution may be associated with the size difference between *TT4* and *TT8*, because a probability of two *tt4* mutants out of 19 candidate mutants was not significant in the binomial test when mutation frequency per DNA length was assumed to be equal. However, all analyzed four mutants harbored extremely large deletions and most of the breakpoints of the deletions were located far from the *TT* genes (Fig. 4). This observation implies that the unequal distribution of the *tt* mutants may be caused by other reasons such as chromosomal location of the marker genes. Further analyses using different marker gene sets are necessary to give a solid explanation for the mutation frequency differences. In this study, because *pab2-1 ban* (*tt4 ban*) and *pab3-1 ban* (*tt8 ban*) were used to detect mutations, two *TT* genes that differed substantially in terms of size (*TT4* and *TT8*) were compared regarding their mutation frequencies. In addition to *tt4* and *tt8*, there are some other *tt* mutants with unique seed colors^24^. For example, *tt9* seeds are tan-like or grayish^34^, whereas *tt19* seeds at the ripening stage are pale brown, but the intensity of the coloration increases after a long-term desiccation^35^. The *TT9* (5794 bp) and *TT19* (1246 bp) genes are similar in size to *TT8* and *TT4*, respectively. Artificial double mutants (i.e., *ban* and other *tt* mutants) that produce seeds with varying colors, making them distinguishable from one another, can be used to construct multiple heterozygous plants for detecting de novo mutations at their *TT* loci and for comparing their mutation frequencies in M_1_ plants. These analyses will help to clarify whether mutation frequencies at specific genomic loci can vary following an ionizing radiation treatment of plants.

## Methods

### Plant materials

The Arabidopsis *ban-4*/*anthocyanin spotted testa* mutant (Columbia (Col-0) accession), which was isolated by Tanaka et al*.* in our laboratory^28^, was used in this study. The *ban-4* mutant, which has a 49-bp deletion in *ANTHOCYANIDIN REDUCTASE*, accumulates anthocyanins (i.e., compounds closely related to proanthocyanidins) in the immature seed coat. Therefore, *ban* has red immature seeds^36,37^. A mutagenized M_2_ population of the *ban* mutant^25^ was used for screening *pab* phenotypes. Another *ban* mutant, *ban* (Nö), which is produced by the insertion of the *Ds* transposon in the Nössen accession (pst13633)^38,39^ and was provided by RIKEN Genomic Sciences Center, was used for the mapping analysis of the newly isolated *pab* mutants. All of the Arabidopsis materials were grown in growth cabinets under standard conditions at 23 °C with a 16 h-light/8 h-dark cycle. Experimental research on plants including the collection of plant material was performed in accordance with relevant institutional, national, and international guidelines and legislation.

### Screening and characterization of *pab* mutants

The coloration of immature seeds was investigated by opening the young siliques of M_2_ individuals under a stereomicroscope (Leica Microsystems, Wetzlar, Germany) at around 6–7 days after flowering. Two mutants (*pab2-1 ban* and *pab3-1 ban*) with *pab* phenotypes that were identified in the mutagenized M_2_ population of the *ban* plants were self-pollinated to obtain their M_3_ seeds. The rough-mapping analysis was conducted using the F_2_ populations of *pab2-1 ban* or *pab3-1 ban* and *ban* (Nö) to evaluate the linkage with the CAPS and simple sequence length polymorphism (SSLP) markers and determine the approximate position of the mutated *pab* genes^29,40^. The candidate genes responsible for the *pab2-1* and *pab3-1* phenotypes (i.e., *TT4* and *TT8*, respectively) were characterized according to a PCR amplification using site-specific primers (Supplementary Table S1). The PCR (40 cycles) was completed using the AmpliTaq Gold 360 polymerase (Thermo Fisher Scientific, Waltham, MA). The amplified fragments for the *TT8* region were analyzed by Sanger sequencing to identify small mutations responsible for the *pab3-1* phenotype.

### Irradiation of double heterozygous plants with carbon ions

Double heterozygous seeds (F_1_ seeds) were obtained by cross-pollinating *pab2-1 ban* and *pab3-1 ban* plants. The seeds were sown on Murashige and Skoog medium containing 2.5% sucrose in plates under sterile conditions. After a 4-day vernalization at 4 °C in darkness, the plates were transferred to a growth cabinet for an incubation at 23 °C under continuous light. Before the mutagenesis experiment, the sensitivity of wild-type Arabidopsis seedlings to different doses of accelerated carbon ions (17.3 MeV/u ^12^C^5+^) was assessed at Takasaki Ion Accelerators for Advanced Radiation Application (TIARA) by calculating the survival rates of plants irradiated at 1 DAG. Plants were considered to have survived the treatment if they had greenish true leaves at 4 weeks after irradiation. A survival curve was drawn according to the single-hit multitarget theory as previously described^41^. In this study, 15 Gy was used for the mutagenesis of the double heterozygous seedlings at 1 DAG. At around 5–7 days after the irradiation, the seedlings were transplanted into a soil mixture comprising vermiculite (Hakugen, Tokyo, Japan) and TM-2 (Takii Seed, Kyoto, Japan) (1:1). They were then incubated in a growth cabinet under standard conditions at 23 °C with a 16 h-light/8 h-dark cycle until flowering and seed formation.

### Screening of anthocyanin-less seeds in the irradiated double heterozygous plants

At the immature seed stage, anthocyanin-less seeds were screened in the irradiated and non-irradiated populations of double heterozygous plants as described for the screening of the *pab* mutant. When anthocyanin-less seeds were not detected in three or more siliques on the first inflorescence stem, the basal region of the first stem was cut and the second and subsequent inflorescence stems were induced to form new siliques from different cell lineages. A total of eight or more siliques from at least three inflorescence stems per plant were analyzed in terms of the color of the immature seeds.

When anthocyanin-less immature seeds were detected in the detached young silique, the corresponding inflorescence stem was allowed to continue to grow to obtain mature seeds in the same lineage as the anthocyanin-less immature seeds, making it possible to compare the mature seed color with that of the parental *pab2-1 ban* and *pab3-1 ban* plants.

Binomial tests^42^ were performed to assess whether a probability for the observed distribution of two mutant types (*tt4*-type and *tt8*-type) was significantly different to an expected probability that mutation frequencies are assumed to be equal, or biased depending on the gene size, between *TT4* and *TT8*.

### PCR analysis using genomic DNA from the seed coat-enriched tissues of anthocyanin-less seeds

To examine the mutations responsible for the anthocyanin-less seed phenotype in the M_1_ generation, we collected the seed coats from the respective mutant seeds because the embryo and endosperm contain the paternal and maternal genotypes in the M_2_ generation, whereas the seed coat is composed of only the maternal genotype^43^. When anthocyanin-less immature seeds were detected in a detached young silique during the screening, the remaining fresh immature seeds in the same silique (about 5–15 seeds) were used in bulk to collect the seed coat cells because they were considered to have the same maternal genotype. The immature seeds were gently pushed on a glass slide with forceps to separate the seed coat from the embryo. The immature seed coats separated from the embryo were pooled in a distilled water droplet, washed gently several times to remove endosperm cells, and transferred to a mortar. All of the dissection steps were completed with samples maintained on ice. The seed coat-enriched tissues were ground in liquid nitrogen using a pestle. Genomic DNA was then extracted from the ground material using the DNeasy Plant Mini kit (Qiagen, Hilden, Germany).

The genomic DNA extracted from the seed coat-enriched tissues served as the template for the site-specific PCR amplification of the *TT4* and *TT8* regions using the same primers as those used for the *pab2-1* and *pab3-1* analyses. The amplicons were purified using the QIAquick PCR Purification kit (Qiagen) and then analyzed by Sanger sequencing.

The *TT4* and *TT8* DNA fragments in the seed coat-enriched DNA were quantified by a qPCR analysis, which was performed using the KAPA SYBR Fast qPCR kit (Kapa Biosystems, Wilmington, MA) and the CFX96 Real-Time System (Bio-Rad, Hercules, CA). A melting curve analysis was performed to confirm the specificity of the amplification by the primers listed in Table S1. The *TT4* and *TT8* DNA fragments were quantified according to the comparative Ct method^44^ and then expressed as the *TT4*:*TT8* ratio, which was normalized against the corresponding ratio in the non-irradiated control plants.

### Dosage analysis

Using 1–5 ng seed coat-enriched DNA, the next-generation sequencing libraries were constructed using the KAPA HyperPlus Library Preparation Kit (Kapa Biosystems) and the IDT for Illumina unique dual indexes (Illumina, San Diego, CA). The pooled libraries were sequenced on the Illumina NovaSeq 6000 and NextSeq 500 platforms, with PE150 chemistry. Low-quality reads and adapter sequences were removed from the raw data using Illumiprocessor (version 2.0.9; https://illumiprocessor.readthedocs.io/en/latest/). The remaining clean reads were mapped to the Arabidopsis reference genome (TAIR10.27; https://www.arabidopsis.org/) using the Burrows–Wheeler Aligner (version 0.7.5; http://bio-bwa.sourceforge.net/), SAMtools (version 1.3.1; http://samtools.sourceforge.net/), and Picard-tools (version 1.119; https://broadinstitute.github.Io/picard/). The obtained BAM files were used for the dosage analysis as previously described^45^, with minor modifications. The bin size was set as 100 kb, and the average read depth in the respective bins was determined using the goleft depth algorithm (https://github.com/brentp/goleft). The read depth in each bin was normalized against the median read depth for all five chromosomes in each line. The resulting RRD values in each bin were plotted to the corresponding chromosomal region.

## Supplementary Information

Supplementary Information.

## Data Availability

The sequencing data have been deposited in DDBJ database (https://www.ddbj.nig.ac.jp/index-e.html) with the accession numbers of DRA014725.
