# Supplementary material for: Development of a simple multiple mutation detection system using seed-coat flavonoid pigments in irradiated Arabidopsis M1 plants
Source: Sci Rep. 2022 Dec 28;12:22467. doi: 10.1038/s41598-022-26989-z (PMC9797493; doi:10.1038/s41598-022-26989-z)
Supplement: Supplementary file 1 — Supplementary Information. [file 41598_2022_26989_MOESM1_ESM.pdf]

Title: Development of a simple multiple mutation detection system using seed-coat flavonoid pigments in irradiated Arabidopsis M_1 plants

Authors: Satoshi Kitamura[1,*], Shoya Hirata[1,2], Katsuya Satoh[1], Rie Inamura[1,2], Issay Narumi[2], and Yutaka Oono[1]

[1] Project "Ion Beam Mutagenesis Research", Department of Radiation-Applied Biology Research, Takasaki Advanced Radiation Research Institute, National Institutes for Quantum Science and Technology, Takasaki, Gunma 370-1292, Japan
[2] Graduate School of Life Sciences, Toyo University, Itakura, Gunma 374-0193, Japan
* kitamura.satoshi@qst.go.jp

A

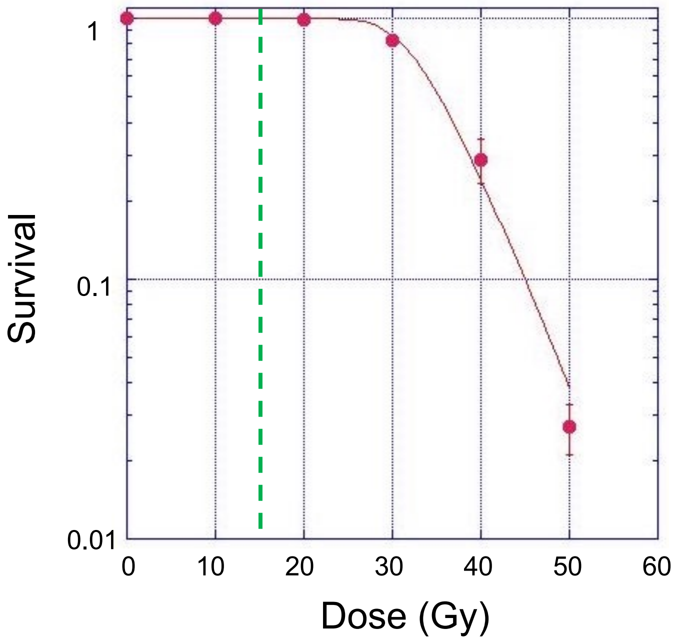

Fig. S1 Survival curve for wild-type seedlings irradiated with carbon ion beams at 1 day after germination. The dose used for mutagenizing seedlings is labeled with a green dotted line (15 Gy). Mean values and standard errors from three biological replicates are plotted.

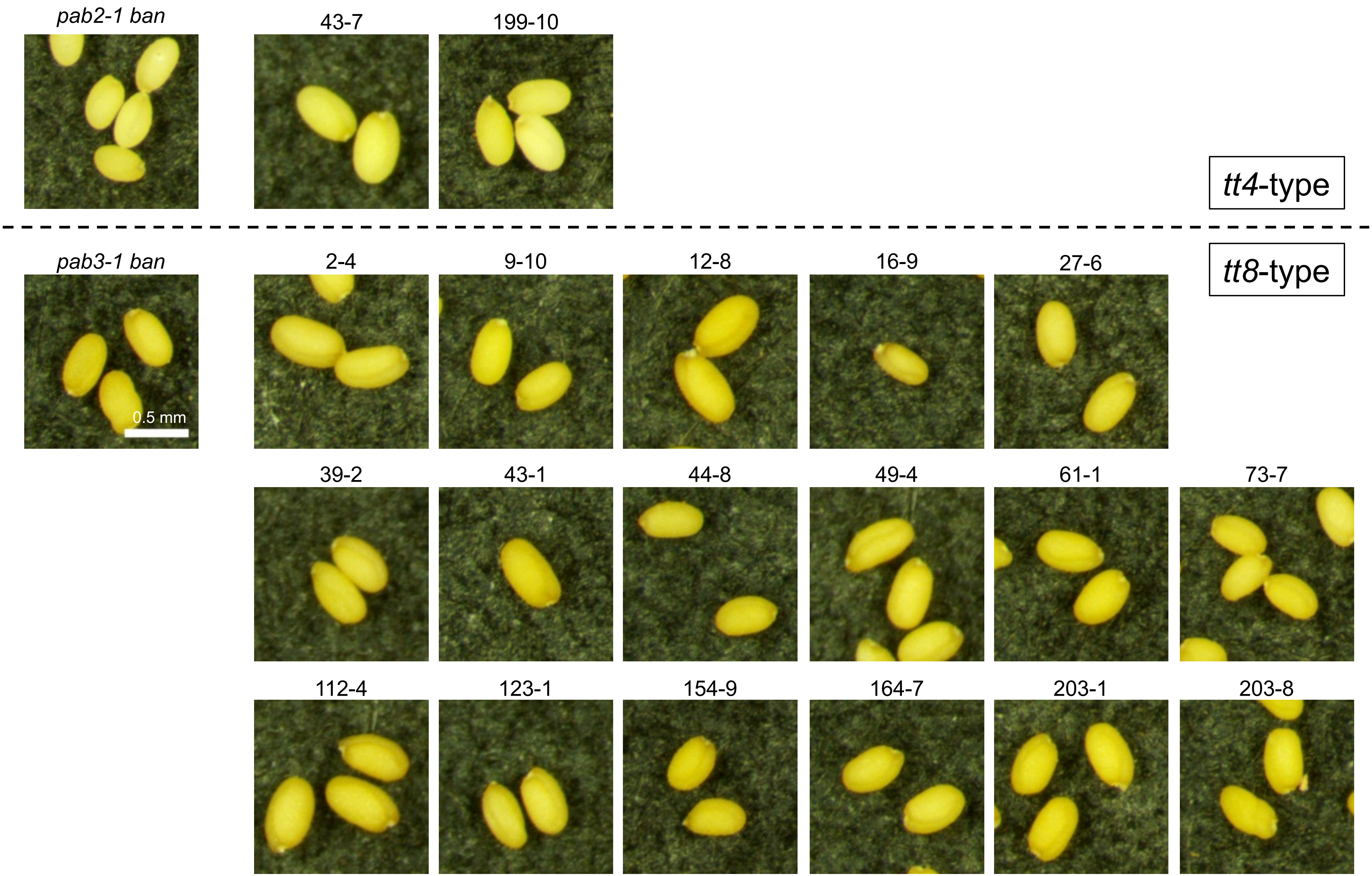

Fig. S2 Mature seed phenotypes for anthocyanin-less mutants resulting from irradiated double heterozygous plants.

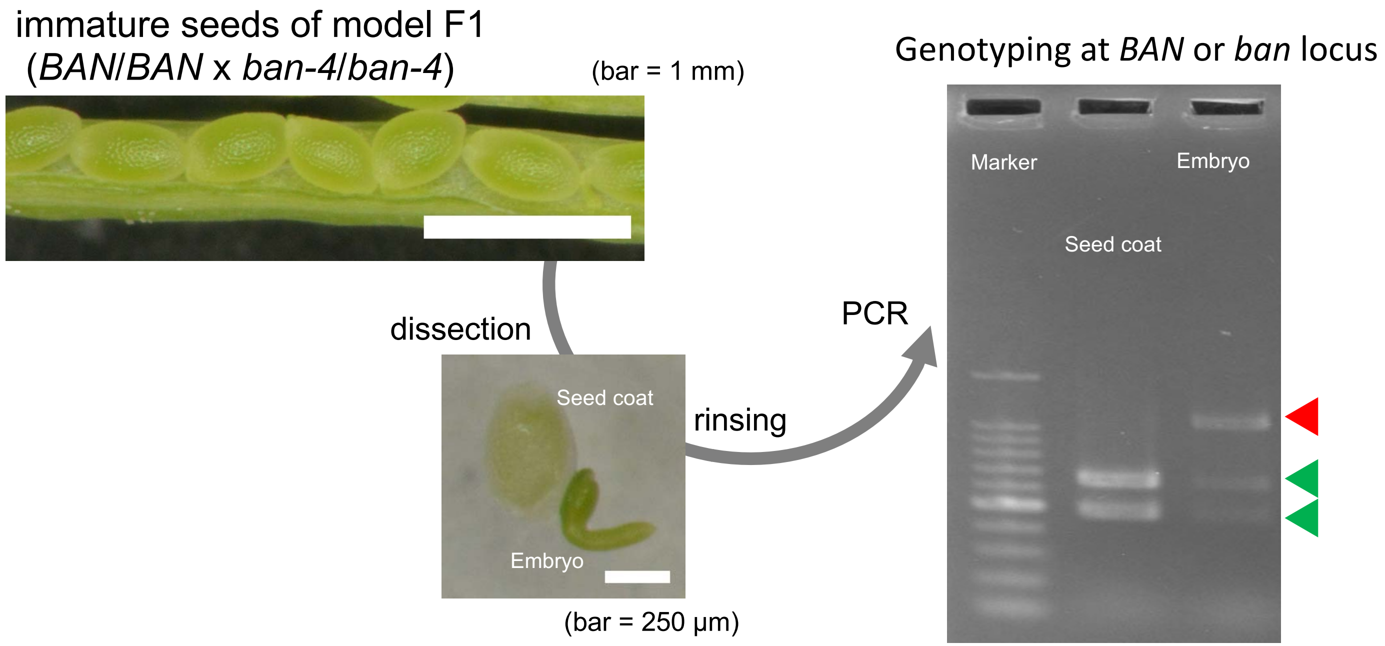

Fig. S3 Analysis of seed coat-enriched DNA using model hybrid seeds (*BAN*/*BAN* stigma with *ban-4*/*ban-4* pollen).

In the model hybrids, the seed coat cells had a maternal *BAN*/*BAN* genotype, whereas the embryo cells had a mixture of maternal and paternal genotypes (*BAN*/*ban-4*). The PCR amplification of the *BAN* and *ban-4* fragments followed by the CAPS analysis with *Scr*FI digestion generated the maternal *BAN* bands (green arrowheads), but not the paternal *ban-4* band (red arrowhead), for the seed coat-enriched DNA. The CAPS band pattern for the dissected embryos is shown as a control.

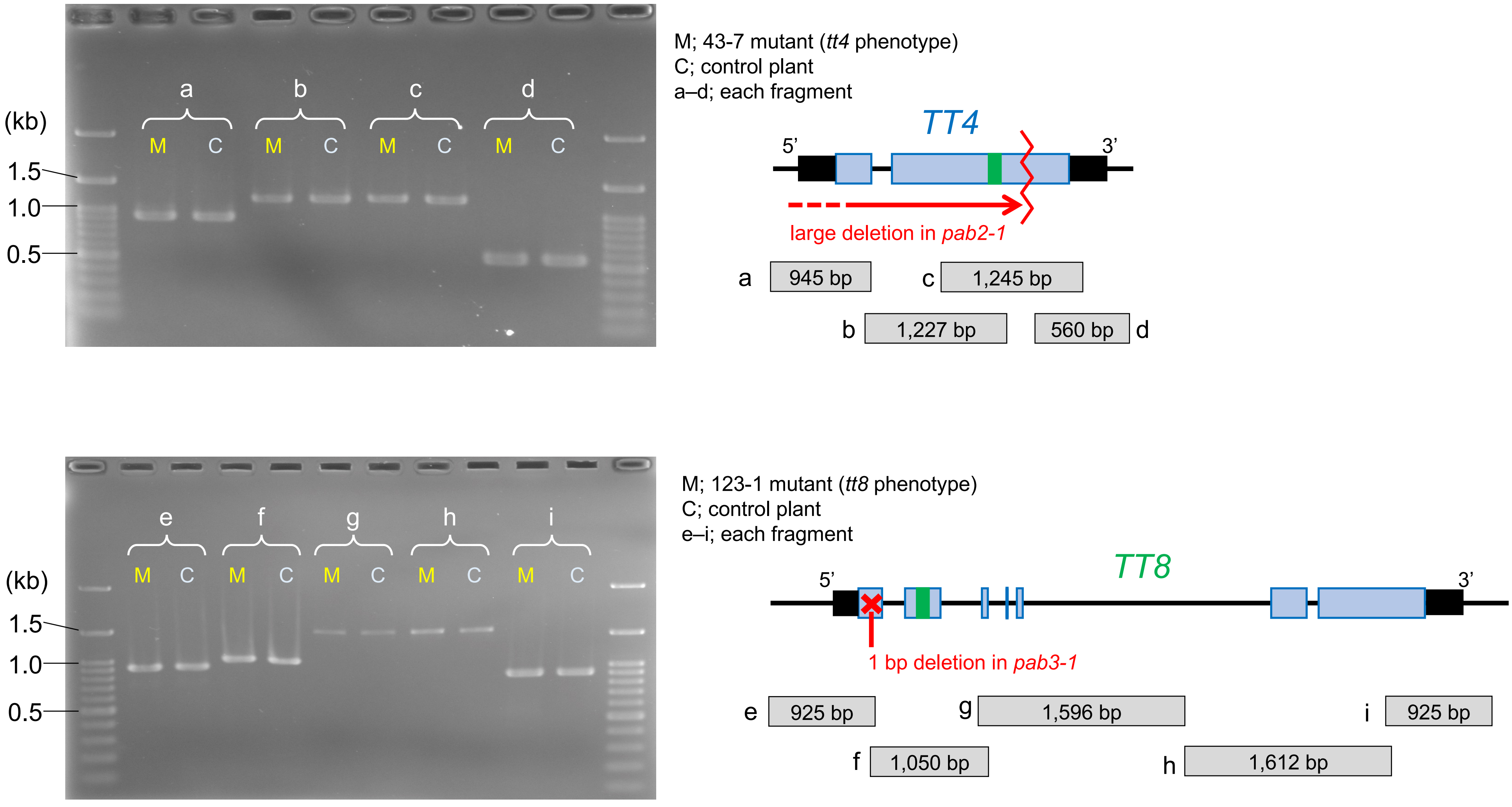

Fig. S4 Representative amplification patterns for the site-specific PCR analysis of *TT4* and *TT8* using DNA from anthocyanin-less seed coat-enriched tissues.

The DNA samples from the mutants (M) and the non-irradiated double heterozygous control plant (C) were used to amplify each fragment (a–i) in *TT4* (top) and *TT8* (bottom). Representative amplification patterns for the *tt4*-type (43-7) and *tt8*-type (123-1) mutants are shown as an example. The location of each fragment and the gene composition are presented in the panels on the right. Pale blue and black boxes represent exons and untranslated regions, respectively. Green bars indicate the region used for the qPCR analysis.

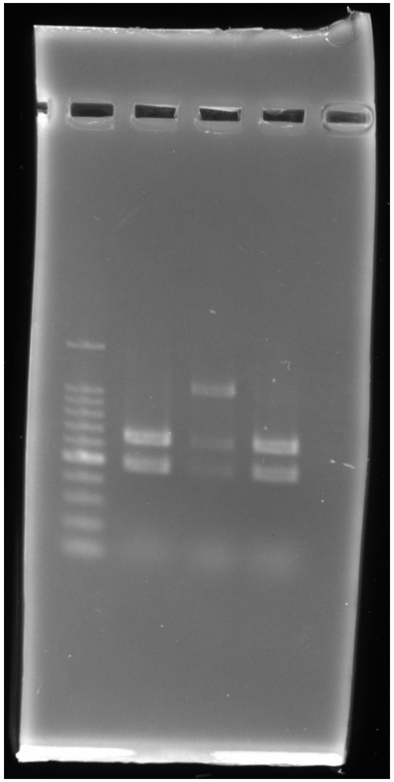

Fig. S5 Uncropped gel images of Fig. S3.

The fourth lane from the left is another seed coat sample.

PCR on *TT4* gene

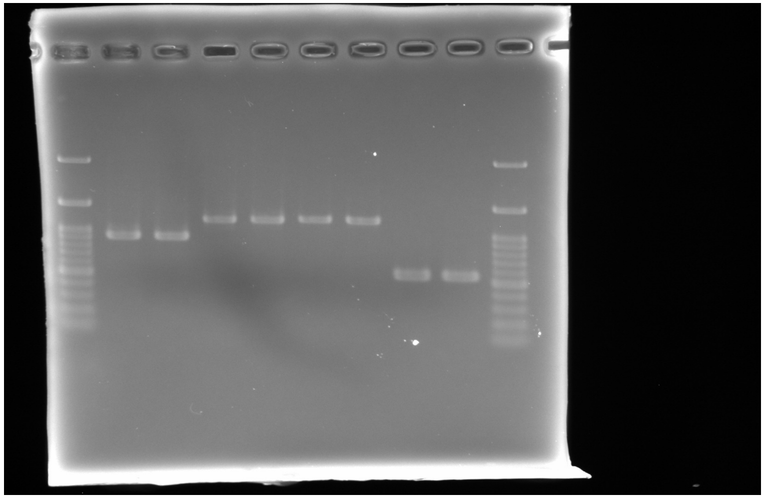

PCR on *TT8* gene

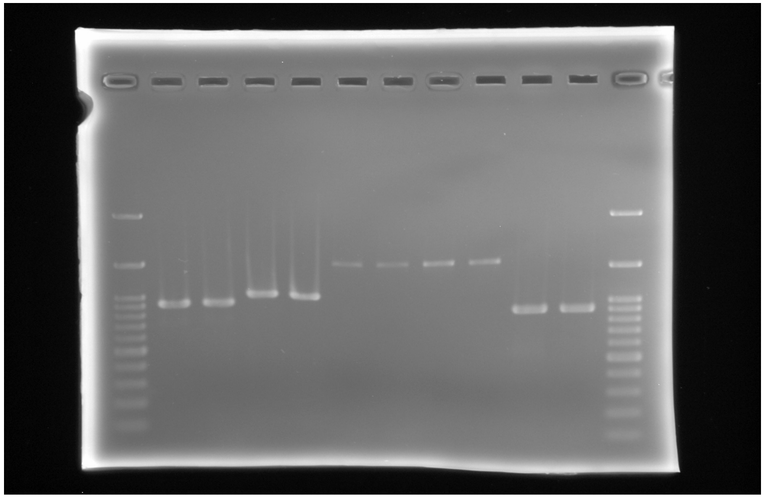

Fig. S6 Uncropped gel images of Fig. S4.

Table S1 Primers used in this study

Primer name	Sequences (5’ -> 3’)	
TT4_f1	TGCATCTTGACATCTTCCGTTG	for fragment a
TT4_r1	ACGTACACATGCGCTTGAACTT	
TT4_f2	TTGGCTATTGGCACTGCTAAC	for fragment b
TT4_r2	GGCAGATAGAAGGCAAGCGTTCTG	
TT4_f3	GATCACTCATGTCGTCTTCTGCAC	for fragment c
TT4_r3	GAAGACAAGTATAGATGCGTGG	
TT4_f4	ACGCAAAACTTTAATCCTGTCTT	for fragment d
TT4_r4	AAGCTGCAATTTCGACGTGG	
TT8_F1	GTGGCTAGTGGCTGCTACTTG	for fragment e
TT8_R1	GTCCAGTCCACAGATTGAACC	
TT8_F2	GGATGAATCAAGTATTATTCCGGCA	for fragment f
TT8_R2	CAACTTCATTTGCACCACTTAGCCA	
TT8_F3	ATGCCAGGAAAAGCGTATGCAAGGA	for fragment g
TT8_R3	CCTGCTTTTACGCTCACGATC	
TT8_F4	GATCGTGAGCGTAAAAGCAGG	for fragment h
TT8_R4	TATTATGACTTGGTGGATGGCTCTT	
TT8_F5	CGGCAGTTCATACCTCGGTGA	for fragment i
TT8_R5	GGAAGTGGAGCCAATGCAGTT	
gqTT4_F	TCACGTGTTGAGCGAGTATG	for real-time PCR (TT4)
gqTT4_R	GTCCGAAACCAAACAAGACACC	
gqTT8_F	AGACGAGGAAGACAACTCAACC	for real-time PCR (TT8)
gqTT8_R	TGCATGCTCTTGCTTCTGAC	
